# StayRose: A photostable StayGold derivative redshifted by genetic code expansion

**DOI:** 10.1016/j.jbc.2025.110832

**Published:** 2025-10-16

**Authors:** Will Scott, Esther Ivorra-Molla, Dipayan Akhuli, Teresa Massam-Wu, Pawel K. Lysyganicz, Rylie Walsh, Matthew Parent, Jonathan Cook, Lijiang Song, Abhishek Kumar, Falk Schneider, Masanori Mishima, Allister Crow, Mohan K. Balasubramanian

**Affiliations:** 1 Centre for Mechanochemical Cell Biology and Division of Biomedical Sciences, Warwick Medical School, University of Warwick, Coventry, UK; 2 Department of Chemistry, University of Warwick, Coventry, UK; 3 Josephine Bay Paul Center, Marine Biological Laboratory, Woods Hole, Massachusetts, USA; 4 School of Life Sciences, University of Warwick, Coventry, UK

**Keywords:** StayRose, StayGold, mCherry, genetic code expansion, 3-aminotyrosine, fluorescence, microscopy, protein engineering, synthetic biology, crystal structure

## Abstract

Photobleaching of fluorescent proteins often limits the acquisition of high-quality images in microscopy. StayGold, a novel dimeric GFP recently monomerized through sequence engineering, addresses this challenge with its high photostability. There is now a focus on producing different colored StayGold derivatives to facilitate concurrent tagging of multiple targets. The unnatural amino acid 3-aminotyrosine has previously been shown to redshift superfolder GFP upon incorporation into its chromophore *via* genetic code expansion. Here, we apply the same strategy to redshift StayGold through substitution of tyrosine-58 with 3-aminotyrosine. The resultant red fluorescent protein, StayRose, shows an excitation wavelength maximum of 530 nm and an emission wavelength maximum of 588 nm. Importantly, the monomeric mStayRose retains the favorable photostability *in vivo* in *Escherichia coli* and zebrafish embryos. A high-resolution crystal structure of StayRose confirms the modified structure of the amino chromophore within an unperturbed 3D fold. Although reliant on genetic code expansion, StayRose provides an important step toward developing redshifted StayGold derivatives.

StayGold is a GFP with extremely high photostability. Engineered from a *Cytaeis uchidae* protein first isolated in 2022, its minimal photobleaching under prolonged excitation makes StayGold valuable in time-lapse microscopy (1, 2, 3, 4, 5). Although the structural basis for its photostability remains unknown, we previously reported the crystal structure of StayGold (2), an 11-strand β-barrel with a chromophore covalently formed by G57–Y58–G59 residues in a central α-helix, alongside a buried chloride ion that interacts with several amino acids within the β-barrel. Different colored photostable StayGold derivatives would allow multitarget imaging over long time frames. Many red fluorescent proteins have one more double bond than green proteins at the N–Cα bond of the first chromophore residue, which extends the π-conjugation system of delocalized electrons to cause a redshift (6). Replacing tyrosine at the superfolder GFP (sfGFP) chromophore (S65–Y66–G67) with an unnatural amino acid, 3-aminotyrosine (Fig. 1*A*), shifts fluorescence into the red range because of a lone electron pair introduced by the amino group (7, 8, 9). Unnatural amino acids can be incorporated into proteins *via* genetic code expansion, which employs orthogonal tRNA synthetases and tRNA to recode the amber stop codon (UAG) for the chosen amino acid (10). We hypothesized that incorporating 3-aminotyrosine into the StayGold chromophore might produce a similar redshift without compromising high photostability.

**Figure 1:**
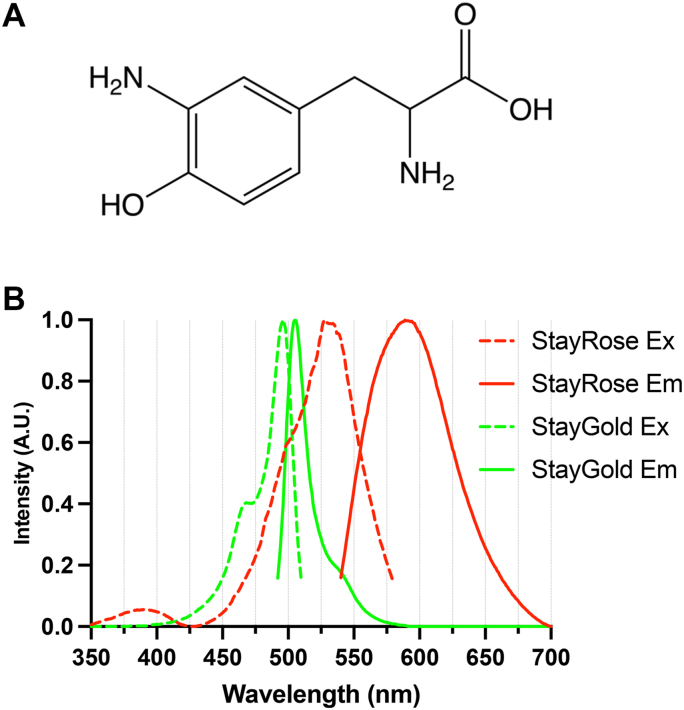
**StayRose isStayGold redshifted *via* 3-aminotyrosine incorporation at Y58**. *A*, structure of 3-aminotyrosine. *B*, comparison of StayRose and StayGold excitation and emission fluorescence spectra. StayRose has an excitation peak of 530 nm and an emission peak of 588 nm, whereas StayGold has an excitation peak of 497 nm and an emission peak of 504 nm. StayRose prep #1 was used for this, and StayGold spectra are from FPbase (1).

## Results

Incorporation of 3-aminotyrosine was trialed at Y58 in StayGold, which is equivalent to Y66 in sfGFP, resulting in visibly red bacterial cultures. His-tagged StayGold bearing 3-aminotyrosine at Y58, which we named StayRose, was purified (Fig. S1), and 3-aminotyrosine incorporation was confirmed *via* mass spectrometry of tryptic peptide fragments (Fig. S2*A*). StayGold exhibits peak excitation at 497 nm and emits fluorescence with a peak at 504 nm (2). StayRose shows peak excitation at 530 nm, accompanied by a small shoulder around 497 nm (Fig. 1*B*), indicating the presence of a minor StayGold fraction from promiscuous incorporation of tyrosine. When excited at 530 nm, StayRose emits red fluorescence with a peak at 588 nm. Four StayRose protein preparations were made, two with 2 mM 3-aminotyrosine supplementation in bacterial cultures (prep #1 and #2) and two with 1 mM (prep #3 and #4). The intensity of the 497 nm excitation peak increased in preparations with lower 3-aminotyrosine concentrations and was comparable to the 530 nm peak (Fig. S3*A*). Excitation of prep #3 (1 mM 3-aminotyrosine) with blue light (488 nm) results in fluorescence peaking at 504 nm, with a tail extending beyond 600 nm (Fig. S3*B*).

The variable fraction of StayGold species between StayRose preps #1 and #3 was further confirmed by mass spectrometry (Fig. S2*B*). A minor peak 15 Da smaller than the main peak corresponds to the tyrosine-derived chromophore in prep #3, with a lower mass than the 3-aminotyrosine-derived variant. In prep #1, another peak, 20 Da larger than the main peak, corresponds to the nascent protein before chromophore formation *via* dehydration (−18 Da) and dehydrogenation (−2 Da). Sample aeration of prep #1 for 84 h removed the nascent peak, hypothesized to be due to completion of chromophore maturation *via* facilitation of oxidation. The size of the nascent peak relative to the main peak also showed preparation-dependent variation. Consistent with this, the peak absorbance/extinction coefficient of StayRose samples, based on the total protein concentration, varied from 2.5 to 3.5 × 10^4^ M^-1^ cm^-1^. The quantum yield (QY) of StayRose fluorescence, excited at 530 nm, was determined using mCherry (QY = 0.22) (11) as the standard. The measured StayRose QY values (0.13–0.34) varied between preparations but compare favorably to the QY reported for 3-aminotyrosine-bearing sfGFP (0.037) (9).

**Figure 2:**
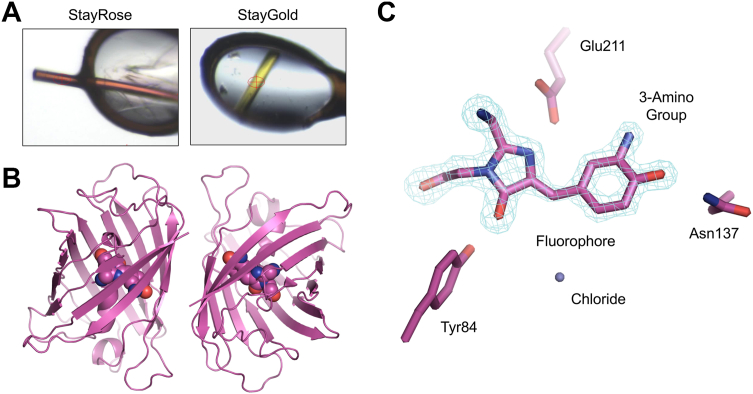
**1.6 Å crystal structure ofStayRose**. *A*, crystals of StayRose and StayGold frozen in loops prior to X-ray data collection. *B*, structure of the StayRose dimer. The chromophore is shown as atomic spheres. *C*, close-up view of the amino-modified chromophore. A 5σ-contoured omit map shows quality of underpinning electron density.

To further characterize StayRose, we determined its crystal structure at high resolution (Fig. 2, Table S1). StayRose crystals are intensely red but otherwise have the same morphology as StayGold crystals (Fig. 2*A*), and the overall structure of StayRose is virtually identical to StayGold (Fig. 2*B*). An omit map showing unbiased density for the amino-modified chromophore confirms that the amino group is well defined, of high occupancy, and locked in a single conformation. The chromophore makes hydrogen bonds with Y64, E211, and N137 residues, but the amino group itself does not form any hydrogen bonds (Fig. 2*C*). The StayRose structure confirms that 3-aminotyrosine is comfortably accommodated by the StayGold fold and that the redshift is due solely to the additional amino group with no further modifications elsewhere in the chromophore. To the best of our knowledge, these are the first structural observations of any 3-aminotyrosine-based chromophore.

StayGold is an obligate dimer, which can affect fusion localization and function. Three studies have reported different monomerizing sequence alterations (2, 12, 13). We reported a single residue change, E138D, that achieved monomerization and maintained photostability (2). StayRose was mutagenized to produce mStayRose(E138D), which was then purified (Fig. S1). The mStayRose(E138D) variant was confirmed to be monomeric by size-exclusion chromatography, whereas StayRose showed dimerization (Fig. S4). Interestingly, the emission spectra of mStayRose(E138D), which showed excitation peaks at 497 nm and 530 nm comparable to StayRose (Fig. S3*C*), did not exhibit an extended red fluorescence tail as was observed for dimeric StayRose under 488 nm excitation (Fig. S3*B*) but otherwise retained similar absorption and emission properties. This suggests that the red tail is likely because of FRET between a minority fraction of StayGold–StayRose heterodimers present within the StayRose sample, which can be resolved by monomerization. Such a mStayGold(E138D) contamination can be seen as the 497 nm peak in Figure S3*C*.

**Figure 3:**
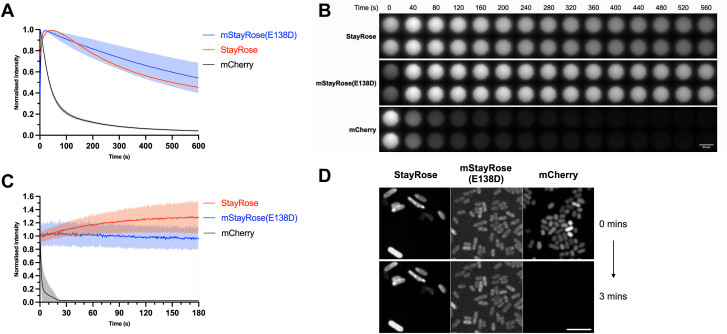
**StayRose andmStayRose(E138D) are highly photostable**. *A*, *in vitro* photostability assays show that StayRose and mStayRose are photostable, and gain intensity during initial periods of laser exposure, whereas mCherry quickly loses intensity. mStayRose(E138D) had repetition variability. Intensity measurements are normalized against peak values for each protein (n = 12). *B*, representative time-lapse images of fluorescent proteins during *in vitro* assays. Intensity is normalized by peak intensity, and the scale bar represents 20 μm. *C*, *in vivo* bacterial photostability assays showed that StayRose and mStayRose(E138D) are photostable, whereas mCherry photobleaches quickly. Intensity measurements are normalized to starting intensity (n = 100 cells). *D*, representative images of *in vivo* assays. Intensity is normalized by peak fluorescence, and the scale bar represents 5 μm. All error bars show standard deviation.

*In vitro* photostability assays of gel-immobilized StayRose and mStayRose(E138D) proteins showed both to be photostable, with only modest reductions in fluorescence intensity observed under laser illumination (Fig. 3, *A* and *B*). StayRose fluorescence was unexpectedly observed to increase during the first minute of laser exposure. This could be related to oxidation and protein maturation induced by laser exposure or represent an unexpected photoactivation property of the 3-aminotyrosine-based chromophore. Purified mCherry (Fig. S1) rapidly lost brightness in the first 100 s, dropping 80% in intensity. As expected, the peak fluorescence intensity of mCherry was brighter than StayRose, influenced by a laser, and a filter set optimized for mCherry. A lower fluorescence intensity was observed for mStayRose(E138D) than StayRose. The E138D mutation is known to slightly lower the QY of StayGold from 0.92 to 0.87 (2), but of the three reported StayGold monomers, mStayGold(E138D) has the highest QY and the second highest molecular brightness (14). *In vivo* bacterial photostability assays provide further evidence for the photostability of StayRose and mStayRose(E138D) (Fig. 3, *C* and *D*), although the expression level of mStayRose(E138D) was far lower than StayRose and mCherry. StayRose shows a gradual increase in fluorescence, mStayRose(E138D) mostly maintains its starting intensity, and mCherry quickly photobleaches. Differences between *in vivo* and *in vitro* photostability likely reflect expression variability in bacteria as well as the two different microscopy setups: constant spot illumination in total internal reflection fluorescence microscopy (TIRFm) (*in vitro* assays) and intermittent spot illumination in spinning disk microscopy (*in vivo* assays).

mStayRose(E138D) is a less perturbing tag than StayRose but exhibits diminished brightness and bacterial expression levels. StayGold itself is known to have low bacterial expression levels, and we are looking into strategies to improve StayGold expression and brightness, including the addition of flanking terminal sequences and further amino acid substitutions. We added short tails to both ends of mStayRose, n1 and c4 sequences, respectively, which improved the brightness of expressing bacteria (Fig. S3*D*), consistent with previous findings in StayGold (12, 14).

It would be beneficial to further redshift the 530 nm excitation peak of StayRose closer to widely used 561 nm lasers. Inspired by the T203Y mutation in sfYFP (15), we found that a K192Y mutation of mStayGold(E138D) results in a 4 nm excitation peak shift to 501 nm and an 8 nm emission peak shift to 512 nm (Figs. S1*A*, S3*E*). Introduction of this mutation into mStayRose(E138D) yielded spectra with peaks comparable to those of the original mStayRose(E138D) (Fig. S3*F*), although the 588 nm emission peak is slightly redshifted from the original 574 nm peak. The K192Y mutation seems to reduce efficiency of 3-aminotyrosine incorporation, with an enlarged 497 nm excitation peak detected.

**Figure 4:**
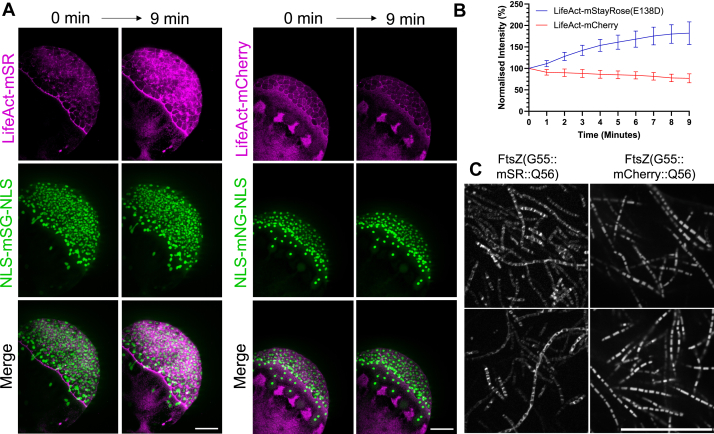
**mStayRose(E138D) facilitates improved imaging in zebrafish embryos and bacteria**. *A*, a zebrafish embryo injected with LifeAct-mStayRose(E138D) (LifeAct-mSR) and NLS-mStayGold(E138D)-NLS (NLS-mSG-NLS) shows an increase in LifeAct-mSR intensity during time-lapse imaging, whilst a zebrafish embryo injected with LifeAct-mCherry and NLS-mNeonGreen-NLS (NLS-mNG-NLS) shows a decrease in LifeAct-mCherry intensity. *B*, quantification of fluorescence intensity in zebrafish embryos shows a photoactivation phenomenon in LifeAct-mSR, whereas LifeAct-mCherry photobleaches. *Error bars* show standard deviation (n = 3). *C*, mStayRose(E138D) (mSR) allows visualization of defined FtsZ rings in *Escherichia coli* with a division phenotype, where overexpression of mCherry fusions obscures clear ring observation. All images are representative, and all scale bars represent 30 μm. NLS, nuclear localization signal.

The employment of mStayRose(E138D) in biological applications was explored. We used zebrafish embryos as a eukaryotic biological model because of their optical clarity and the ability to inject purified proteins into the embryos, which are approximately 400 μm in diameter. Actin cytoskeleton and nuclei were imaged in live zebrafish embryos using injected recombinant LifeAct-mStayRose(E138D) and nuclear localization signal NLS-mStayGold(E138D)-NLS, with clear actin and nuclear structures visible, respectively (Fig. 4*A*). Time-lapse acquisitions of these labels were compared against embryos with LifeAct-mCherry and NLS-mNeonGreen-NLS (Fig. 4, *A* and *B*, S1*C*. mStayRose(E138D) grows in intensity as imaging progresses, supporting trends observed in bacteria and *in vitro* (Fig. 3), in contrast to the decrease seen in mCherry fluorescence. Having established the photostability of mStayRose in a eukaryotic organism, we next tested its usability in bacteria. To this end, mStayRose(E138D) and mCherry (a routinely used red-fluorescent protein) were inserted into a loop of the bacterial ring protein, FtsZ, at a position used in a previous study (16). In live *Escherichia coli*, these fusion proteins create a faulty division phenotype in which the cells become vastly elongated with many visible rings (Fig. 4*C*). The reduced efficiency in expression of genetically expanded proteins improves imaging in this scenario, allowing visualization of FtsZ rings with mStayRose(E138D) at a definition that mCherry overexpression obscures. All proteins used in Figure 4 employed the flanking regions tested in Figure S3*D*.

## Discussion

In summary, we have modified the StayGold chromophore with 3-aminotyrosine to create a new fluorescent protein, StayRose, with significantly redshifted excitation and emission spectra. We obtained a high-resolution X-ray structure of StayRose, showed that it retains the high photostability of StayGold, and monomerized it using the E138D mutation. The monomer was subsequently engineered with flanking regions to improve bacterial expression, and targeted mutagenesis was trialed to further redshift it. We demonstrated applicability of this monomer, through fusion to proteins of interest and imaging in live bacteria and injected zebrafish embryos. In future, we will generate transgenic zebrafish expressing the appropriate orthogonal tRNA–tRNA synthetase from *E*. *coli*, to allow direct incorporation of 3-aminotyrosine into mRNA encoding mStayRose through translation *in vivo* and to avoid injection of proteins that may be difficult to purify as mStayRose fusions from *E*. *coli*.

Several additional steps are needed to bring StayRose to the point it can be used as an effective fluorescence imaging tool. There are known limitations associated with genetic code expansion–based tools, such as the requirement for multiple components reducing ease of use. Some studies have addressed problems using single plasmid expression constructs (17). The presence of low levels of StayGold because of tRNA synthetase infidelity is a problem for multitarget imaging studies. We have demonstrated that this can be modulated using increased 3-aminotyrosine concentrations; however, it may be difficult to eliminate fully. Improvement of 3-aminotyrosine incorporation efficiency would make StayRose a more powerful tool. This could be achieved *via* sequence engineering of the 3-aminotyrosine-tRNA synthetase, with enhancement quantified from measurement of red *versus* green fluorescence in StayRose preparations, with a higher value pointing to increased fidelity of 3-aminotyrosine incorporation. StayRose shows a photoactivation phenomenon that warrants further examination of the electron distribution and maturation chemistry underpinning 3-aminotyrosine-based fluorophores. A similar, albeit minor photoactivation has been observed in (n2) OxStayGold (c4) (18). Purposeful misincorporation of tyrosine *via* reduced 3-aminotyrosine supplementation, as observed in Figure S3*A*, will enable expression of a protein of interest as a mixture of fusions with the green or the red form of mStayRose(E138D) from a single construct. This can be used to assess the monomeric *versus* oligomeric state of the fusion target *in vivo* or *in vitro* through detection of FRET produced by red–green oligomers. StayRose demonstrates that the structural features crucial for StayGold’s photostability are not limited to green fluorescence, marking an important step toward developing photostable StayGold variants in a spectrum of colors.

## Experimental procedures

### Cloning, expression, and purification of fluorescent proteins

A description of all plasmids used in this work can be found in Table S2. pET-MCN-10His-StayRose and pET-MCN-10His-mStayRose(E138D) plasmids were generated by substituting Y58 with an amber stop codon (TAG) *via* mutagenesis of pET-MCN-10His-StayGold (Addgene #211360) and pET-MCN-10His-mStayGold(E138D) (Addgene #211361), respectively. For site-directed mutagenesis, Q5 High Fidelity DNA polymerase (NEB; M0491) was used to PCR amplify constructs with primers complementary except for the desired mutation. The products of this were gel extracted with the QIAquick Gel Extraction Kit (Qiagen; 28704) and chemically transformed in DH5α *E*. *coli* for screening of colonies *via* DNA extraction with the QIAprep Spin Miniprep Kit (Qiagen; 27104), restriction digestion, and sequencing. pET21a-6His-mStayGold(E138D + K192Y) plasmid was generated *via* cloning of mStayGold(E138D) from pET-MCN-10His-StayGold into a pET21a vector, followed by K192Y mutagenesis. K192Y mutagenesis was also used to produce pET-MCN-10His-mStayRose(E138D + K192Y) from pET-MCN-10His-mStayRose(E138D). Several fluorescent fusion proteins with His tags were cloned into a pET-MCN vector *via* Gibson assembly of synthetic G-blocks (IDT), namely LifeAct-(n1)mStayRose(E138D) (c4), LifeAct-mCherry, NLS-(n1)mStayGold(E138D) (c4)-NLS, NLS-mNeonGreen-NLS, FtsZ(G55::(n1)mStayRose(E138D) (c4)::Q56), FtsZ(G55::mCherry::Q56), and (n1)mStayRose(E138D) (c4). LifeAct fusions used a GSGSG linker to connect the proteins. The SV40 NLS fusions used a GSGSG to fuse NLS onto the fluorescent protein N terminus and a GS linker to fuse an NLS to the C terminus. Two-residue SG and GS linkers were used on either side of the fluorescent protein inserted into FtsZ. Also used was pBAD-6His-mCherry (AddGene #54630). For incorporation of 3-aminotyrosine in all genetic code expansion experiments, pEvol-MjaYRS (AddGene #153557) plasmid was used. All sequences were confirmed *via* nanopore whole plasmid sequencing.

mCherry, StayRose, mStayRose(E138D), mStayGold(E138D + K192Y), LifeAct-(n1)mStayRose(E138D) (c4), (n1)mStayRose(E138D + K192Y) (c4), LifeAct-mCherry, NLS-(n1)mStayGold(E138D) (c4)-NLS, and NLS-mNeonGreen-NLS proteins were expressed and purified using N-terminal His tags. For this, each of the fluorescent protein reporter constructs were transformed into BL21(DE3) *E*. *coli*, with a further transformation of pEvol-MjaYRS plasmid carried out for those requiring 3-aminotyrosine incorporation. Lysogeny broth (LB; aka Luria–Bertani) liquid cultures were grown up shaking at 36 °C. For cultures requiring 3-aminotyrosine incorporation, 100 mM 3-aminotyrosine (Bachem; 4027898) in 0.2 M HCl was added to a final concentration of either 1 mM or 2 mM 3-aminotyrosine in an absorbance of 0.2 at 600 nm cultures. An absorbance of 0.6 at 600 nm liquid cultures was induced for 28 h at 18 °C with 0.5% arabinose and/or 0.9 mM IPTG as appropriate. Resulting bacterial pellets were lysed *via* sonication in lysis buffer (50 mM sodium phosphate buffer, 300 mM NaCl, 0.1 mM MgCl_2_, 10 mM imidazole, 0.3 mM PMSF, and cOmplete protease inhibitor cocktail [pH 7.5]). HisPur Ni–NTA resin (Thermo Scientific; 88222) was used to purify proteins in wash buffer (50 mM sodium phosphate buffer, 500 mM NaCl, 30 mM imidazole [pH 7.5]) before elution in elution buffer (50 mM sodium phosphate buffer, 500 mM NaCl, 500 mM imidazole [pH 7.5]). Sample buffer was exchanged using PD midiTrap G-25 desalting columns (Cytiva; 28918008) to storage buffer (20 mM Hepes and 150 mM NaCl [pH 7.5]). Purified sfGFP, StayGold, and mStayGold(E138D) protein samples used in this study were the same preparations used in the study by Ivorra-Molla *et al*. (2).

### Digested protein mass spectrometry analysis

Purified protein samples were run on a 12% SDS-PAGE acrylamide gel and Coomassie blue staining, from which bands were excised. Bands were destained in 50% ethanol and 50 mM ammonium bicarbonate (3 × 20-min washes at room temperature), dehydrated in ethanol (5 min at room temperature), and reduced in 40 mM chloroacetic acid and 10 mM Tris(2-carboxyethyl)phosphine (5 min at 70 °C). Following further washing in 50% ethanol and 50 mM ammonium bicarbonate (3 × 10-min washes at room temperature) and dehydration in ethanol (5 min at room temperature), gels were hydrated in 2.5 ng/μl trypsin diluted in trypsin resuspension buffer (Promega; V5111), and incubated overnight at 37 °C. The liquid fraction was collected, and further peptide was extracted from the gel *via* sonication in a water bath (3 × 10 min at room temperature) in 25% acetonitrile and 5% formic acid with liquid fraction collection after each round. Combined liquid fractions were concentrated by evaporation in a speed vacuum and resuspended in 50 μl 2% acetonitrile and 0.1% trifluoroacetic acid.

Tryptic peptides were separated *via* reversed-phase chromatography. Subsequent mass spectrometric analysis utilized two C18 columns on a NanoElute UHPLC system (Bruker Daltonics): a 75 μm × 40 cm 1.9 μm Bruker NanoElute Forty Analytical column and an Acclaim PepMap μ-precolumn cartridge 300 μm i.d. × 5 mm 5 μm 100 Å (Thermo Fisher Scientific). Mobile phase buffer A was 0.1% formic acid in water, and mobile phase buffer B was 0.1% formic acid in acetonitrile. Following μ-precolumn equilibration with 2% aqueous acetonitrile containing 0.1% formic acid, samples were loaded and peptides were eluted at 350 nl min^-1^ onto the analytical column by increasing the concentration of mobile phase B from 3% B to 15% across a 31 min time frame, then to 35% B across 10 min, and to 85% B across 3 min, followed by a 0% B re-equilibration for 10 min. A hybrid timsTOF Pro (Bruker Daltonics) online was paired with NanoElute through a CaptiveSpray nano-electrospray ion source (19). Data-dependent parallel accumulation-serial fragmentation mode was used to operate the timsTOF Pro. Peptides were separated by ion mobility conditional on charge states and collisional cross sections. The method settings were: ion mobility range 1/K_0_ start 0.6 Vs/cm^2^ end 1.6 Vs/cm^2^, duty cycle 100%, ramp rate 9.42 Hz, and mass range 100 to 1700 *m/z*. Peptide datasets were searched against protein-specific databases using Mascot Database Search by Matrix Science, with up to two missed cleavages, ±20 ppm and ±0.6 Da peptide tolerance, fixed modification of cysteine carbamidomethylation, and variable modification of methionine oxidation, as well as UAA modification where applicable.

### Whole protein mass spectrometry analysis

Purified StayRose and mStayRose(E138D) protein samples (100 μg) were buffer exchanged in a 10 kDa cutoff 0.5 ml Amicon centrifugal filter device to 500 μl 35 mM ammonium acetate (pH 7.4). Mass spectrometry analysis was carried out using a Bruker MaXis II coupled with Dionex 3000RS UHPLC with C4 column (100 × 2.1 mm, 2.7 μm), a gradient of 5% to 100% water/acetonitrile, and a flow rate of 0.2 ml/min. The matured sample was aerated for 84 h prior to mass spectrometry.

### Structure determination using X-ray crystallography

Crystallization screens used 12 mg/ml StayRose in 150 mM NaCl and 20 mM Hepes (pH 7.5) in MRC 2-drop plates. Sitting drops were set using a Formulatrix NT8 robot with 1 μl drops of 1:2 or 2:1 protein solution and crystallization reagent. Crystals were obtained in the SG1 HT96 Eco Screen (Molecular Dimensions) condition H4 (25% PEG 3350, 0.1 M Bis–Tris [pH 6.5], and 0.2 M sodium acetate).

StayRose crystals were cryoprotected with 30% glycerol. X-ray diffraction experiments were conducted remotely at beamline I04 at the Diamond Synchrotron. Diamond’s autoprocessing pipeline integrated diffraction images using Dials (20). The CCP4 suite (21) was used to perform subsequent processing, with AceDRG (22) used to generate constraints for the 3-aminotyrosine ligand. Aimless (23) was used for scaling and space group determination. Phases were determined by molecular replacement using Phaser (24). Model building and rebuilding used Coot (25) and refinement was performed with REFMAC (26). The model was validated using tools from PROCHECK (27), Rampage (28) and Coot (25). Images were generated using PyMOL (29). The StayRose structure was deposited in the Protein Data Bank with accession code 9G7Q.

### Fluorescence excitation and emission spectra

A Cary Eclipse fluorescence spectrophotometer with a 5 nm excitation and emission slit width was used to acquire fluorescence spectra. Spectra of three serial (undiluted, twofold, and fourfold) dilutions of each protein were taken using a 600 nm min^-1^ scan rate at 1 nm intervals.

### Extinction coefficient determination

A Cary 50 Conc UV–visible spectrophotometer was used to measure absorbance of each protein at four serial (100%, 75%, 50%, and 25%) dilutions. The molar extinction coefficients (ε_FP_) were calculated using the peak absorbance (A_peak_), the absorbance at 280 nm, and the theoretical extinction coefficients at 280 nm (ε_280_) (with one tyrosine omitted from the sequence, https://web.expasy.org/protparam/) (30).

$$\varepsilon_{\text{FP}}=\varepsilon_{280}\times A_{\text{peak}}/A_{280}$$

### QY measurements

Fluorescent protein QYs were determined by comparing the ratio between the integrated fluorescence emission and the absorbance at the peak excitation for the fluorescent protein, with the same ratio for mCherry (29). The reported QY value for mCherry of 0.22 was used for this (11). The fluorescence/absorbance ratios were each determined using the gradient from a plot of integrated fluorescence *versus* absorbance.

### Size-exclusion chromatography

An ÄKTA Pure FPLC with a Superdex 75 Increase 10/300 GL column was used to perform size-exclusion chromatography, with a buffer of 20 mM Hepes and 150 mM NaCl (pH 7.5) and a 0.8 ml min^-1^ flow rate. For each run, ∼1 mg ml^-1^ (38 μM) protein was loaded onto the column *via* a 0.5 ml loop. As well as the novel fluorescent proteins, additional runs of bovine serum albumin (66 kDa) and hen egg white lysozyme (14 kDa), StayGold (52 kDa [as a dimer]), mStayGold(E138D) (26 kDa), mCherry (31 kDa), sfGFP (27 kDa), and Bio-Rad molecular weight standards (thyroglobulin [670 kDa], γ-globulin [158 kDa], ovalbumin [44 kDa], myoglobin [17 kDa], and vitamin B12 [1.35 kDa]) were used for estimation of protein molecular weights.

### Bacterial microscopy

Liquid cultures of BL21(DE3) *E*. *coli* were transformed with the fluorescent protein expression constructs described above (and pEvol-MjaYRS plasmid where appropriate), treated with 3-aminotyrosine (if required), and induced as per the steps described in the *Protein purification* section. Induced bacteria were imaged on microscopy slides, using a spinning disk confocal microscope (Andor Revolution XD imaging system, equipped with a Nikon ECLIPSE Ti inverted microscope, a confocal unit Yokogawa CSU-X1, an Andor iXon Ultra 888 EMCCD camera, a 100× oil immersion 1.45 numerical aperture (NA) Nikon Plan Apo Lambda objective [69 nm/pixel], and Andor IQ acquisition software). Cells were imaged for 3 min at 0.5 s intervals with a 60% power (1.19 mW) 561 nm laser. The microscope area of illumination is 11,748.7 μm^2^, corresponding to an overall illumination of 10.1 W cm^-2^. A set area of fluorescence intensity from each cell was measured from every slice using the image processing software Fiji (https://imagej.net/software/fiji/). Intensity measurements were normalized to the first data point.

For imaging of fluorescent FtsZ fusions in *E*. *coli*, cells were cultured and induced as above and imaged on an Andor Revolution XD CSU-X1 Yokogawa spinning-disk system with a Nikon ECLIPSE Ti inverted microscope, an Andor iXon Ultra EMCCD camera, a Nikon Plan Apo λ 100×/1.45 NA oil-immersion objective lens (80 nm/pixel), and Andor IQ3 software.

### *In vitro* photostability

Photostability of purified proteins was measured using the method described in the study by Cranfill *et al*. (29). In brief, fluorescent proteins were embedded in a polyacrylamide gel (20% acrylamide [mono:bis 29:1], 150 mM NaCl, and 20 mM Tris [pH 7.5]), which was polymerized between a 1.5 mm coverslip and a glass slide with 15.5 μm polystyrene beads (Bangs Laboratory; PS07N) as spacers. The samples were observed using a TIRFm setup, namely a Nikon Eclipse Ti-E/B microscope equipped with a Ti-E TIRF illuminator (561 nm CW laser line), a Zyla sCMOS 4.2 camera, a 100× oil immersion 1.49 NA Nikon CFI Apo TIRF objective lens, and Andor iQ3 software. A 381.9 μm^2^ octagonal area within each sample was illuminated using a 561 nm laser beam at 100% intensity and a 0° angle of incidence with 990 ms exposure (plus a 10 ms dark interval for image acquisition) repeated at 1 s intervals for 10 min. The laser power was measured as 347 μW, corresponding to illumination at 90.9 W cm^-2^.

### Zebrafish embryos

Zebrafish work was approved by the Institutional Animal Care and Use Committee and the US Public Health Service Policy on Humane Care and Use of Laboratory Animals. Zebrafish were housed at the Marine Biological Laboratory aquatic facility under a 14:10 h light:dark cycle at 28.5 °C following Zebrafish International Resource Center guidelines. Natural spawning was used to collect embryos, which were then maintained in E3 media unfed. Proteins (1 nl 50 μM in PBS) were injected into zebrafish embryos at the 1-to-8-cell stage using a PV830 injector set-up. Embryos were dechorionated with 1 mg/ml pronase (PRON-RO; Merck) and mounted in 1% low melting agarose on a glass bottom dish (P50G-1.5-30-F; MatTek). All zebrafish embryo works were carried out prior to 5 days postfertilization.

A custom-built stage-scanning line confocal microscope was used to capture large-area images of zebrafish embryos at high resolution. The system is configured in dual-view geometry (both upright and inverted) for imaging samples in a Petri dish or glass slide. For this particular application, an upright illumination and imaging path was utilized for both excitation and detection. Four commonly used lasers (405 nm, 488 nm, 561 nm, and 640 nm) are combined *via* dichroic filters and passed through an acousto-optic tuneable filter for intensity modulation and fast on/off switching. The beam is spatially cleaned using a lens–pinhole pair, and an iris is employed to truncate the Gaussian profile and use the central region for more uniform illumination.

To generate line illumination, the beam is shaped using a Powell lens (3 mm input aperture, 45° fan angle). The resulting line profile is collected by a spherical lens and projected into the back aperture of the illumination objective (25 × 1.1 NA water or 25 × 1.05 NA silicone), creating a diffraction-limited line at the sample plane. Fluorescence excited along the line is collected by the same objective, passed through a quad-band dichroic and emission filter set, and focused onto an sCMOS camera (PCO Edge 5.5) *via* a 200 mm tube lens to form a line image. Instrument control and data acquisition are handled through a custom MATLAB-based graphical user interface. To maximize speed, the system reads a narrow region of interest (16-pixel rows at the center of the sCMOS chip) repeatedly as the sample is translated in one-pixel steps using an ultrasonic piezo linear X–Y stage (Physik Instrumente; U751.24). These line images (“stripes”) are computationally assembled postacquisition into a full 2D plane by shifting the stripes appropriately. The process is then repeated in the Z plane by stepping the illumination objective with a piezo stage (Physik Instrumente; PIFOC P-725.4CDE2) to generate 3D image stacks. Multicolor imaging is achieved either by sequential acquisition of color planes or by alternating excitation wavelengths stripe by stripe. All datasets are saved in TIFF format with full metadata, enabling visualization and analysis in both commercial and open-source software.

mStayGold(E138D) and mNeonGreen fusion proteins were excited with a 488 nm laser at consistent laser intensity, whereas mStayRose(E138D) and mCherry fusion proteins were excited with a 561 nm laser. Z-stacks (75 × 1 μm slices) were taken every minute for 9 min. Two to five equally sized areas from each embryo (n = 3) were selected for fluorescence intensity analysis *via* a Fiji macro (https://github.com/fcefwe/2025_06_27_Fiji_Macro_Photobleaching). Intensity measurements were normalized to the first data point.

## Data availability

All datasets are included either within the article or the supporting information.

## Supporting information

This article contains supporting information (2, 26, 28, 31).

## Conflict of interest

The authors declare that they have no conflicts of interest with the contents of this article.
