# Supplementary material for: StayRose: A photostable StayGold derivative redshifted by genetic code expansion
Source: J Biol Chem. 2025 Oct 16;301(12):110832. doi: 10.1016/j.jbc.2025.110832 (PMC12661434; doi:10.1016/j.jbc.2025.110832)
Supplement: Supplementary File 2 [file mmc2.docx]

**Supporting Information**

Supplemental Table

- Table S1. Crystallographic data collection and refinement statistics
- Table S2. List of plasmids used in this work

Supplemental Figures

- Figure S1. His-tagged fluorescent protein purification
- Figure S2. Mass spectrometry analyses of purified StayRose and mStayRose(E138D)
- Figure S3. Fluorescence of novel StayGold derivatives
- Figure S4. mStayRose(E138D) is monomeric and StayRose is dimeric

Protocol – Purify 10His-StayRose protein from *E. coli*

**Supplementary Tables**

**Table S1. Crystallographic data collection and refinement statistics.**

|  | **StayRose (9G7Q)** |
| --- | --- |
|  |  |
| **Data collection** |  |
| Beam line | Diamond I04 |
| Wavelength (Å) | 0.95373 |
|  |  |
| **Crystal parameters** |  |
| Space group | P6_1_ |
| Unit cell dimensions (Å) | 133.31, 133.31, 58.35 |
| Unit cell angles (°) | 90, 90, 120 |
|  |  |
| **Reflection data^*^** |  |
| Resolution range (Å) | 44.63-1.65 (1.68-1.65) |
| Unique reflections | 70,220 (3,349) |
| *R_sym_* | 0.150 (1.423) |
| *R_pim_* | 0.033 (0.335) |
| I/σ(I) | 14.3 (2.0) |
| CC_½_ | 0.999 (0.784) |
| Completeness (%) | 98.5 (95.4) |
| Multiplicity | 20.7 (18.3) |
| Wilson B (Å^2^) | 15.8 |
|  |  |
| **Refinement^†^** |  |
| Resolution (Å) | 43.67 – 1.65 |
| Number of reflections | 66,707 |
| *R_overall_* | 0.155 |
| *R_free_* | 0.177 |
| Rms (bond lengths) (Å) | 0.011 |
| Rms (bond angles) (°) | 1.76 |
|  |  |
| **Model B-factors** |  |
| StayGold (chain A) (Å^2^) | 19.5 |
| StayGold (chain B) (Å^2^) | 19.3 |
| Waters (Å^2^) | 30.6 |
|  |  |
| **Ramachandran statistics^‡^** |  |
| Favoured (%) | 98.3 |
| Allowed (%) | 1.7 |
| Outlier (%) | 0 |
|  |  |

Values in parentheses indicate the highest resolution bin.

Refinement statistics are from REFMAC.^26^

Ramachandran statistics as reported by Rampage.^28^

**Table S2. List of plasmids used in this work.**

| Plasmid | Description |
| --- | --- |
| pET-MCN-10His-StayRose | Bacterial expression and purification of StayRose. |
| pET-MCN-10His-mStayRose(E138D) | Bacterial expression and purification of mStayRose(E138D). |
| pET-MCN-10His-StayGold | Bacterial expression and purification of StayGold (AddGene #211360). |
| pET-MCN-10His-mStayGold(E138D) | Bacterial expression and purification of mStayGold(E138D) (AddGene #211361). |
| pET21a-6His-mStayGold(E138D+K192Y) | Bacterial expression and purification of mStayGold (E138D+K192Y). |
| pET-MCN-10His-mStayRose(E138D+K192Y) | Bacterial expression and purification of mStayRose(E138D+K192Y). |
| pET-MCN-10His-LifeAct-(n1)mStayRose(E138D)(c4) | Bacterial expression and purification of LifeAct-mStayRose(E138D) with n1 and c4 linkers. |
| pET-MCN-10His-LifeAct-mCherry | Bacterial expression and purification of LifeAct-mCherry. |
| pET-MCN-10His-NLS-(n1)mStayGold(E138D)(c4)-NLS | Bacterial expression and purification of NLS-mStayGold(E138D)-NLS with n1 and c4 linkers. |
| pET-MCN-10His-NLS-mNeonGreen-NLS | Bacterial expression and purification of NLS-mNeonGreen-NLS. |
| pET-MCN-FtsZ(G55::(n1)mStayRose(E138D)(c4)::Q56) | Bacterial expression of FtsZ with mStayRose(E138D) inserted between G55 and Q56 with n1 and c4 linkers. |
| pET-MCN-FtsZ(G55::mCherry::Q56) | Bacterial expression of FtsZ with mCherry inserted between G55 and Q56. |
| pET-MCN-10His-(n1)mStayRose(E138D)(c4) | Bacterial expression and purification of mStayRose(E138D) with n1 and c4 linkers. |
| pBAD-6His-mCherry | Bacterial expression and purification of mCherry (AddGene #54630). |
| pEvol-MjaYRS | Bacterial expression of tRNA and tRNA synthetase for 3-aminotyrosine incorporation (AddGene #153557). |

**Supplemental Figures**

**Figure S1. His-tagged fluorescent protein purification.** (A) Image of purified fluorescent proteins loaded on PD midiTrap G-25 columns during buffer exchange. Bacterial expression of mStayRose(E138D) was observed to be lower than StayRose, hence the lower intensity colour. (B) SDS-PAGE gel of purified fluorescent proteins used throughout this work. Genetic code expansion commonly produces two side products: a protein truncated at the amber stop codon site and a protein bearing a natural amino acid at the amber stop codon site. The former is due to a low rate of failed amber suppression and the latter due to a low level of recognition for tyrosine by the orthogonal tRNA synthetase. mCherry appears as two bands because of the double bond introduced in the main peptide chain by the matured chromophore, which breaks during sample heating prior to SDS-PAGE. This leaves immature protein at the larger full-sized band, and damaged mature protein as the lower band.^31^ (C) SDS-PAGE gel of purified fluorescent fusion proteins used in Fig. 4A-B.


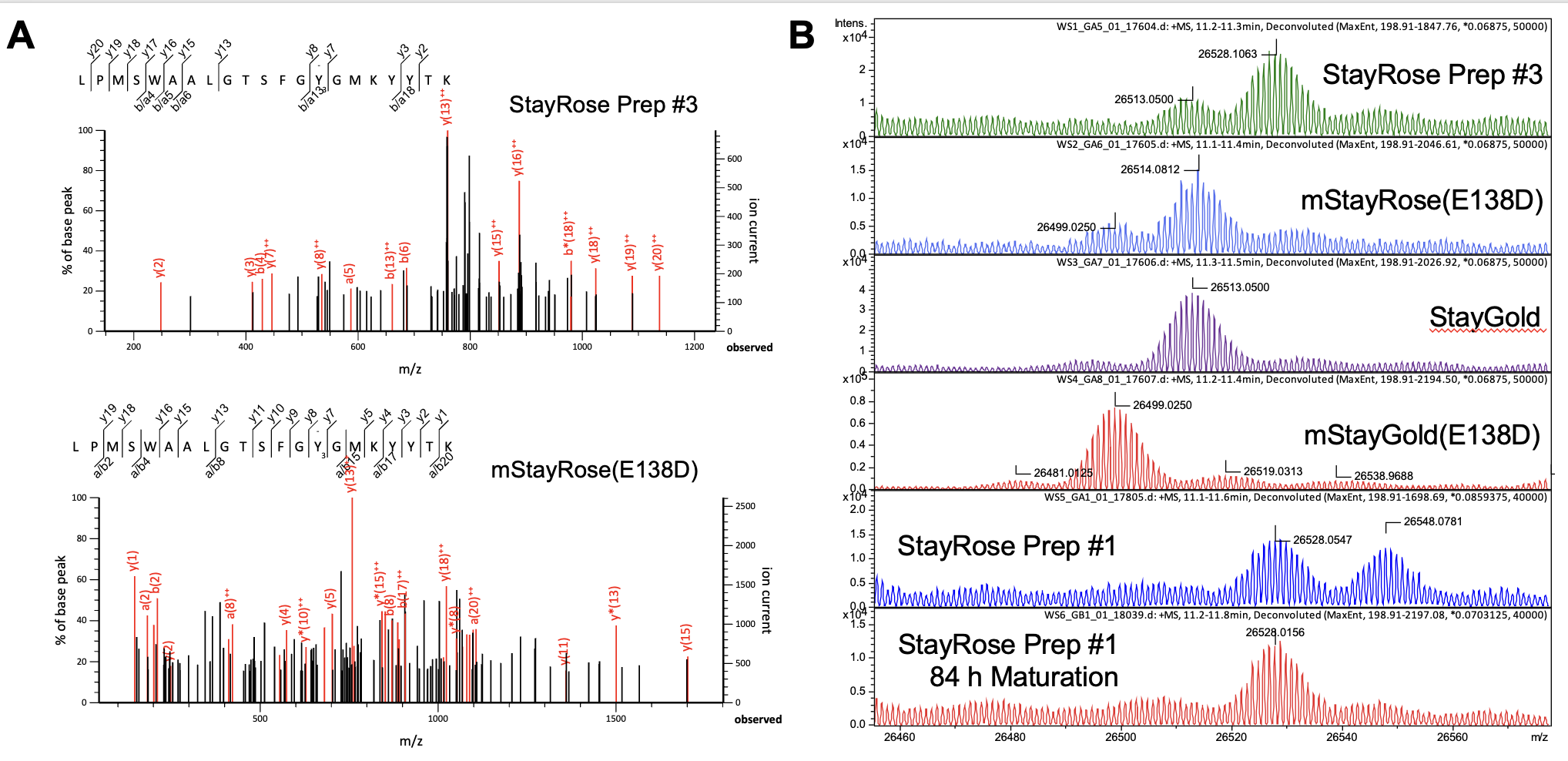


**Figure S2. Mass spectrometry analyses of purified StayRose and mStayRose(E138D).** (A) Spectra of digested StayRose and mStayRose(E138D) peptide fragments confirm 3-aminotyrosine incorporation. (B) Spectra of whole StayRose and mStayRose(E138D) proteins allow quantification of molecules incorporating 3-aminotyrosine (26528 peak for StayRose and 26514 peak for mStayRose(E138D)) or tyrosine (26513 peak for StayRose and 26499 peak for mStayRose(E138D)) at position 58, as well as comparison of nascent (26548 peak) and matured (26528 peaks) protein peaks. The ratio of each varies between preps, indicating variability in incorporation rate.

**Figure S3. Fluorescence of novel StayGold derivatives.** (A) Excitation spectra of four different StayRose protein preparations show variability in 3-aminotyrosine incorporation efficiency. Preparations that used 1 mM 3-aminotyrosine in bacterial cultures have a prominent 497 nm peak, indicating tyrosine misincorporation, whereas preparations with 2 mM 3-aminotyrosine cultures did not. Emissions at 588 nm were measured. (B) Emission spectra with excitation at 488 nm of StayRose Prep #3 shows a lengthier tail beyond 600 nm than mStayRose(E138D). (C) Excitation and emission spectra of StayRose (Prep #1) and mStayRose(E138D). For excitation spectra, 588 nm emissions were detected, and emission spectra were detected at 530 nm excitation. (D) (n1)mStayRose(c4)(E138D) has higher cellular brightness than mStayRose(E138D) in *E. coli* (n = 90 cells). (E) Excitation and emission spectra of mStayGold(E138D + K192Y) and mStayGold(E138D). For mStayGold(E138D + K192Y), 512 nm emissions were detected for excitation spectra, and emission spectra were detected at 501 nm excitation. mStayGold(E138D) spectra from Ivorra-Molla *et al*. (2023)^2^ were used. (F) Excitation and emission spectra of mStayRose(E138D + K192Y) and mStayRose(E138D). 588 nm emissions were detected for excitation spectra, and absorbance at 530 nm for emission spectra. The excitation spectra of mStayGold(E138D + K192Y) (collected at 512 nm emissions) is also shown for reference. For all spectra in this figure, n = 3.

**Figure S4. mStayRose(E138D) is monomeric and StayRose is dimeric.** (A) StayRose elutes earlier than mStayRose(E138D) during size exclusion chromatography, suggesting a larger molecular size. The small mStayRose(E138D) peak at 9 mL is associated with protein aggregates from the protein preparation method. (B) Comparison to size exclusion chromatography standards gives mStayRose(E138D) and StayRose molecular weights of 26 kDa and 52 kDa respectively, implying the former is monomeric and the latter is a dimer.

**Protocol – Purify 10His-StayRose protein from *E. coli***

1. Transform BL21(DE3) *Escherichia coli* (or another appropriate protein expression bacterial strain), with pET-MCN-10His-StayRose (KanR) and pEvol-MjaYRS (ChlR) plasmids on an LB Kan^+^ Chl^+^ agar plate.
2. Inoculate a 2 mL LB Kan^+^ Chl^+^ liquid culture shaking overnight at 37°C.
3. Add the overnight culture to 500 mL LB liquid culture and incubate shaking at 37°C.
4. At OD_600_ 0.2, supplement with 100 mM 3-aminotyrosine (Bachem, 4027898) to a final concentration of 2 mM 3-aminotyrosine.
5. At OD_600_ 0.6, induce with 0.5% arabinose and 0.9 mM IPTG.
6. Incubate shaking for 28 h at 18°C.
7. Pellet cells at 6000 g and resuspend in 10 mL lysis buffer (50 mM sodium phosphate buffer, 300 mM NaCl, 0.1 mM MgCl_2_, 10 mM imidazole, 0.3 mM PMSF and cOmplete™ Protease Inhibitor Cocktail (pH7.5)).
8. Add 150 µL 1 mg/mL lysozyme (Sigma-Aldritch, L6876) and incubate on ice for 30 minutes.
9. Lyse bacteria via sonication (or alternative lysing method).
10. Pellet at 60,000 g for 2 minutes at 4°C.
11. Purify the supernatant on HisPur Ni-NTA resin (Thermo Scientific, 88222), as per the manufacturer’s instructions, with:
    1. Wash buffer: 50 mM sodium phosphate buffer, 500 mM NaCl, 30 mM imidazole (pH 7.5).
    2. Elution buffer: 50 mM sodium phosphate buffer, 500 mM NaCl, 500 mM imidazole (pH7.5).
12. Buffer exchange the proteins using PD midiTrap G-25 desalting columns (Cytiva, 28918008), as per the manufacturer’s instructions, to storage buffer (20 mM HEPES and 150 mM NaCl (pH 7.5)).
13. Snap freeze and store at -80°C.
