# Supplementary material for: StayRose: A photostable StayGold derivative redshifted by genetic code expansion
Source: J Biol Chem. 2025 Oct 16;301(12):110832. doi: 10.1016/j.jbc.2025.110832 (PMC12661434; doi:10.1016/j.jbc.2025.110832)
Supplement: Supplementary File 3 [file mmc3.pdf]

JBC JOURNAL OF BIOLOGICAL CHEMISTRY

Mass Spectral Proteomics Methods and Data Check list

This checklist is intended to help authors submit manuscripts that present mass spectral proteomics methods, data and figures in a transparent manner that is compatible with JBC guidelines and best practices in the field, and facilitates reproducing the study. More detailed instructions are available at: Molecular and Cellular Proteomics Guidelines.

A. Source and Isolation of Biological Samples

- ✔ Organism (human or other)
- ✔ Number of technical replicates
- ✔ Number of biological replicates
- ✔ Cell/tissue lysis and fractionation conditions, including how samples were protected from proteolysis, dephosphorylation etc.
- ✔ Protein or peptide enrichment conditions (i.e. immunoprecipitation, affinity methods for PTMs (i.e. phosphorylation or other)
- ✔ Proteolytic digestion conditions, time, temperature, protease type and specificity
- ✔ Peptide clean up and off-line chromatography methods

B. Mass Spectrometry (MS) Conditions (LC-MS or MALDI-TOF-MS)

- ✔ Instrument name (vendor/model), including HPLC and mass spectrometer
- ✔ Details on HPLC column(s), description of mobile phases, flow rate, gradients etc.
- ✔ MS parameters including detailed conditions for data-dependent analysis (DDA), data-independent analysis (DIA) or MALDI MALDI-TOF-MS/MS applications. Including all MS1 and MS2 (or higher-MS3) parameters, resolution, instrument settings etc. For example, included MS/MS scans resolution and dynamic exclusion conditions, collision energy and type.
- ✔ Methods of peptide/protein quantification (i.e. TMT, ITRAQ, SILAC or other) or label -free quantification (i.e. MaxQuant).

C. Data analysis and processing methods

- ✔ Name of software and methods for generating peak lists.
- ✔ Database searching software and version/date
- ✔ Peptide exclusion conditions, number of unique and razor peptides
- ✔ Database(s) used including name, version and taxonomy
- ✔ Fixed and variable modifications, number of missed cleavages allowed, precursor and product ion mass tolerances

ASBMB JOURNALS

☐ Calculation of false discovery rate (FDR) and criteria for acceptant of peptide and protein assignments (i.e. Benjamini–Hochberg method)

D. Statistics and data validation

☐ Type of software or name of program used, including version

☐ Data clustering conditions (if applicable)

☐ Citation of literatures sources used

☐ Methods to determine significance, power analysis or test(s) applied (i.e. modified T-tests, etc.)

E. Data reporting

☑ A supplementary file in Excel format is recommended, which should include:

Protein data

☑ Should include protein name, accession number and molecular weight.

☑ Number of assigned spectra and unique sequences used for protein identification.

☑ Percent sequence coverage, database search score

Peptide data

☑ Include peptide sequence data with start and stop residue numbers, observed mass, mass error. Scores/expect values for assignment, post-translational modifications, probability determination for modification sites

☑ The complete MS proteomics data set must be deposited to a publicly available database (e.g. ProteomeXchange Consortium via the PRIDE partner repository, MASSive); details of how to access the data must be provided in the manuscript.

F. Predictions from Proteomics Data and presentation

☐ Pathway analysis platforms used (i.e. Ingenuity Pathway Analysis (IPA), RoKAI, Kinase-library.phosphosite.org others).

☐ Generation of kinome pathways (Coral, Kinome Render)
